# Grid aware electrification for decarbonising port logistics based on a case study from Sweden

**DOI:** 10.1038/s41598-025-25136-8

**Published:** 2025-11-04

**Authors:** Sankar Mangalath Ramasan, Jagruti Thakur, Sivapriya M. Bhagavathy, Björn Laumert

**Affiliations:** 1 Department of Energy Technology, KTH Royal Institute of Technology, Stockholm, Sweden; 2 PNDC, University of Strathclyde, Glasgow, UK

**Keywords:** Energy infrastructure, Energy storage, Renewable energy

## Abstract

Electrification is emerging as a key strategy for decarbonisation of shore-side energy demand at ports. However, this electrification, particularly involving electric shore-side vehicles (ESVs), has a significant impact on the local electricity grid. A key research gap pertains to the specific challenges of ESV load integration into the grid and the effectiveness of mitigation strategies like smart charging and renewable energy integration at the operational level within ports. This study directly addresses this gap through several key contributions: firstly, by quantifying the impact of ESV loads on a localised port electricity grid; secondly, by introducing and evaluating smart charging strategies coupled with solar photovoltaic (PV) integration; and thirdly, by providing practical insights derived from a real-world case study at the port of Oskarshamn. Key findings include: (i) an impact analysis demonstrating that unmanaged (‘dumb’) ESV charging imposes the highest stress on the local grid, necessitating costly immediate upgrades; (ii) the demonstration that optimized charging significantly reduces grid stress, effectively deferring the need for substantial infrastructure investment; and (iii) the confirmation that solar PV integration further aids in managing peak loads and enhancing overall grid stability and energy independence. These results underscore the efficacy of smart charging and renewable integration in managing ESV loads and improving grid resilience. Furthermore, the study highlights potential pathways for future energy efficiency enhancements and even the possibility of energy export within port systems.

## Introduction

Efforts to achieve global climate targets, as outlined in the Paris Agreement and pursued through initiatives like the European Green Deal, are driving rapid decarbonisation across sectors^1^.

Ports, serving as major nodes for international trade and transport, are pivotal but complex environments for implementing these goals, particularly concerning electricity usage and emissions management^2,3^. Initiatives like FuelEU Maritime^3^ and pilot projects such as TranZero^4^ highlight the sector’s commitment to electrification, including shore-side equipment as a strategy for reducing fossil fuel dependence and greenhouse gas emissions^5^. Port operations, involving the movement of cargo and the use of machinery such as cranes, terminal movers, forklifts, and loaders, have traditionally relied on diesel-powered equipment. Recent years have seen a growing trend toward electrification, driven by the extensive knowledge and experience gained from electrifying heavy-duty and passenger vehicles. Electrification offers faster decarbonization potential and is increasingly seen as a viable solution for port-side applications.

However, the electrification of port utilities poses significant challenges for the distribution capacity of the electricity grid. Grids, designed for historical demand, struggle with the peak loads from electric vehicle (EV) charging, risking substation overloading^6^. Additionally, EV chargers can introduce power quality issues, such as harmonic distortion, affecting transformers^7^. Mitigation measures, such as load management strategies and charge scheduling, have been successfully implemented in passenger EVs, demonstrating economic benefits^8^ and grid loss reductions^7^.

The literature around maritime decarbonisation has looked extensively into both on-shore and off-shore activities. While off-shore decarbonization technique mainly constitutes renewable fuels for ship propulsion^9^, authors have also looked into battery-electric propulsion for the ship^10^ and off-shore charging stations to tackle range limitations^11^. Electrification has proven to be a method to decarbonise onshore activities^12^, and to improve air quality around the port^13^. A case study conducted over different ports in the US shows the full electrification of port has a potential of over 60% GHG emission reduction^14^. The study emphasises on the increased demand on the grid due to both cold ironing and electrified cargo handling equipment and drayage trucks. In another study^15^, the author claims that electrification of ground vehicles, such as the trucks, forklifts and straddle carriers helps achieve broader decarbonisation results. The author focuses on the electrification of cargo handling equipment and the infrastructural upgradation through fast chargers, on-site EVs and battery energy storage, to accommodate the additional demand. A recent handbook on port electrification developed by Pacific Northwest National Laboratory (PNNL), among the other things, suggest that managed charging helps in the better electric fleet integration, in order to manage the required chargers and hence the project cost^16^.

Optimisation techniques, however, has been extensively studied in the passenger EV space. A study shows that unpredictable loads are induces on the utility grid and suggests mathematical optimisation models as a mitigation technique^17^. A similar mathematical optimisation, using Mixed Integer Linear Programming (MILP), has been implemented as a case study in a neighbourhood in Stockholm, as an effort to reduce grid losses and reduced cost of electricity import^7^. Another study presents an optimization model for allocating slow and fast chargers at electric boat stations in Stockholm, emphasizing tailored infrastructure planning and showing investment payback within seven years. However, the study does not consider the impact on the electricity network, limiting insights into grid integration challenges^18^. Furthermore, introducing optimal charge scheduling in Electric Bus Rapid Transit (BRT) in Cairo to helped reduce the annual energy cost, representing its applicability in predictable usage environment^19^.

The existing research broadly covers maritime decarbonisation needs and the studies on port electrification, focusing on techno-economic assessments rather than feasibility studies. Optimisation technique, on the other hand, has been well researched within passenger EV space to integrate EV loads. A significant gap persists regarding detailed studies on the operational-level challenges of integrating ESV loads into existing port infrastructure. Furthermore, studies evaluating mitigation strategies frequently consider them in isolation of ESVs operating within a port environment. This lack of detailed, empirically validated assessment of combined ESV load impacts and integrated mitigation strategies like smart charging with solar PV, specifically tailored to port operations with variable loads (e.g., 2–11 h/day) constitutes a significant gat that needs to be addressed. This study addresses this crucial research gap by moving beyond theories and using real operational data where possible. It examines the integration of ESVs and the effectiveness of mitigation strategies within the real-world operational context of the Port of Oskarshamn, a port actively transitioning. The contributions of this paper are specifically tailored to bridge the aforementioned literature weaknesses:

- Firstly, by **quantifying the real operational impact** of ESV charging loads on the local distribution grid based on actual port schedules and usages.
- Secondly, by **introducing and evaluating** the effectiveness of specific smart charging strategies, explicitly considering the operational reality depicted (e.g., varied charging requirements), potentially integrated with solar PV energy sources.
- Thirdly, by providing **empirical**,** real-world insights** derived directly from this on-site agreement for study and real data collected, moving beyond theoretical assumptions.

The focus of this study is to analyse the technical impacts of ESV electrification on the local grid and evaluating the effectiveness of mitigation strategies. An in-depth examination of the associated cost implications and competitive advantages is deemed out of scope for this research paper. The findings underscore the efficacy of smart charging and renewable integration (like the installed PV) in managing ESV loads, deferring infrastructure investments, and enhancing critical requirements like grid stability and operational reliability. These results offer practical and validated information for port operators and grid planners undertaking electrification transitions.

This paper is structured as follows: Sect. “Methodology” presents the methodology which is then implemented for Oskarshamn port as a Case Study in Sect. “Case study” followed by Assumptions in Sect. “Assumptions”, Results and Discussion in Sect. “Results and discussion”, and Conclusion in Sect. “Conclusion”.

## Methodology

**Fig. 1:**
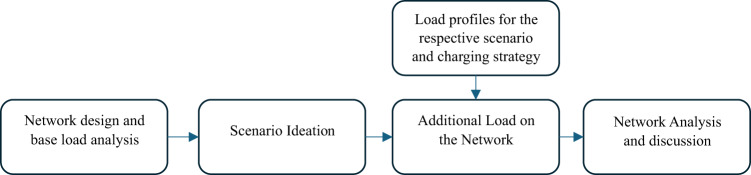
Methodology overview.

The overall methodology is as illustrated in Fig. 1. It involves creating a digital twin of the existing grid, establishing a baseline network state through load flow analysis, ideating scenarios for the integration of Electrified Shore-side Vehicles (ESVs), developing ESV load profiles, and analyzing the impact of different charging strategies on the grid, including dumb charging, load management startegy, and load management strategy with solar PV integration.

### Grid design

The methodology begins with designing the grid using a digital twin of the existing network. This digital twin is created using the Pandapower library for Python. The components modeled in the network include buses, lines, switches, transformers, and loads. The data for designing the grid include the specification all the components used, like the bus voltages, cable length and material, transformer ratings and connected load, and the data is gathered from the network operator. The specifications of these components are matched to the available data values:

- **Buses**: Represent the voltages at each point in the network (in kV).
- **Lines**: Represent the interconnections between buses, including distances and cable parameters.
- **Switches**: Determine whether specific transformers are connected to or disconnected from the network.
- **Transformers**: Step down voltages from the substation to the utility connections, placed according to their location in the network and available specifications.
- **Loads**: Represent the utilities connected to the secondary side of the transformers.

### Baseline network state

A baseline network state is established through load flow analysis of the existing loads connected to the transformers in the grid. This analysis provides network parameters such as voltage levels, power flows, and power losses, which serve as a baseline for comparison with future load changes. Hourly load profiles from the utilities are converted to 15-minute profiles, assuming the same peak demand across each hour, to ensure a more granular representation of the load dynamics.

### Scenario ideation

Scenario ideation provides a structured approach to effectively replacing conventional shore-side vehicles with ESVs. The ideation process considers the decarbonization strategy applicable to a specific port. The number of ESVs connected to the grid during charging varies based on the scenario, impacting the grid accordingly. It is crucial to create ESV load profiles that are added to the existing base load according to the scenario.

### Load profile development

ESV load profiles are developed based on the working patterns of the replaced conventional vehicles and their associated charging parameters. The data for load profile development is gathered from the port authority. This include both the ESV specification, including the battery capacity and charge/discharge rates and the operational characteristics of the replaced shoreside vehicle. The working hours of ESVs vary depending on their operational area within the port and vehicle type. All ESVs undergo overnight slow charging, while some require daytime fast charging to prevent complete battery depletion during operation. Fast charging is scheduled during available breaks, such as machine operator breaks for breakfast, lunch, or coffee. The charging strategy ensures that all ESVs complete their designated daily working hours without depleting the battery completely.

### Charging strategies

Load profiles are created for different charging strategies:

- **Dumb Charging**: This strategy, also referred to as unmanaged or mindless charging, determines the impact of integrating ESVs into the grid without considering network limitations.
- **Load management strategy**: This strategy optimizes the charging schedule to minimize the maximum demand at each timestep, ensuring efficient use of charger availability and reducing the impact on the grid. This is further explained in Sect. “Load management strategy”.
- **Load management strategy with Solar PV Integration**: This strategy integrates solar PV into the network without energy storage, aiming to achieve higher self-sufficiency. The optimization ensures that the grid impact is minimized while maximizing the use of renewable energy.

#### Load management strategy

Load management strategy introduces a systematic approach to optimize the use of resources to balance supply and demand. Optimization strategies for charging use various algorithms to manage the demand, duration and interval of charging. These algorithms help the charging pattern to align with specific goals like peak load management in the grid management, electricity prices or availability of renewable energy sources. While the end-user is often familiar with smart chargers with IoT and real-time control and monitoring, an objective algorithm that works at the back end defines the purpose of optimization.

A Mixed Integer Linear Programming (MILP) based optimization model is created to minimize the total charging demand at each timestep as the objective function. The model is created using the PuLP library for Python along with the GUROBI solver. Various other constraints are defined in terms of maximum charge capacity.

##### Algorithm

The optimization aims to reduce the total charging demand on the external grid at a particular timestep. The model is compiled for a week, considering separate constraints for the weekdays and weekends. This weekly data is then compiled for a year.

The objective function for the optimization is given by the following equation:

$$\:{Minimize\:\:\:P}_{max}=max\sum\limits_{i=1}^{N}{p}_{i}\left(t\right)\:\:\:t\in\:\{\text{0,1},..T\}$$

*where*:

*P*_max_ is the maximum total charging power across all time steps.

*N* is the total number of ESVs.

*p*_*i*_(*t*) is the charging power of ESV *i* at time step *t*.

*T* is the total number of time steps.

##### Constraints

The state of Charge (SOC) in the first time period of the model for each electric vehicle (ESV) is considered full battery capacity.

 

$$\:{SOC}_{i,0}=\:{B}_{i}\quad\forall i$$

*where*:

SOC: State of Charge of the battery in kWh.

SOC_*i*,0_: Initial SOC of ESV *i* in kWh.

*B*_*i*_: Battery capacity of ESV *i* in kWh.

The SOC of each ESV at time *t* + 1 depends on the SOC at time *t*, the charging power, and the discharging rate.

$$\:{SOC}_{i,t+1}=\:{SOC}_{i,t}+\left({U}_{i,t+1}\:.{p}_{i,t+1}.\varDelta\:t\right)-\:\left({d}_{i}\:.{v}_{i,t+1}.\varDelta\:t\right)\:\forall\:i,\forall\:t$$

*where*:

SOC_*i, t*_: State of Charge of ESV *i* at time step *t*, in kWh.

*u*_*i, t*+1_: Binary indicator that equals 1 if ESV *i* is charging at time step *t* + 1, and 0 otherwise.

*p*_*i, t*+1_: Charging power for ESV *i* at time step *t* + 1, given in kW.

∆*t*: Time interval between successive time steps.

*d*_*i*_: Discharge rate of ESV *i*, given in kWh/h.

*v*_*i, t*+1_: Binary indicator that equals 1 if ESV *i* is discharging at time step *t* + 1, and 0 otherwise.

A vehicle cannot charge and discharge simultaneously.

$$\:{u}_{i,t}+\:{v}_{i,t}\le\:1\:\forall\:i,\forall\:t\:$$

The charging power of each ESV is limited by the maximum charger power.

$$\:{p}_{i,t+1}\le\:{u}_{i,t+1}.\text{min}\left({P}_{charger},\:{P}_{i,max}\right)\:\forall\:i,\forall\:t\:$$

*where*:

*P*_charger_: The maximum power that the charger can provide.

*P*_*i*,max_: The maximum charging power that ESV *i* can accept.

The SOC must remain within the battery capacity limits.

$$\:{B}_{i}.{SOC}_{min\%}\le\:{SOC}_{i,t+1}\le\:{B}_{i}\:\:\:\forall\:i,\forall\:t$$

*where*:

*B*_*i*_: Battery capacity of ESV *i*.

SOC_min%_: Minimum SOC as a percentage of the battery capacity.

∀*i*,∀*t*: For all ESVs *i* and all time steps *t*.

The number of ESVs charging simultaneously is limited by the available charging points during fast charge periods.

$$\:\sum\limits_{i}{u}_{i,t}\le\:{n}_{fast}\:\:\forall\:t$$

*where*:

*n*_fast_: Number of available fast charging points.

The total charging power across all ESVs at any time step must not exceed *P*_max_.

$$\:\sum\limits_{i}{p}_{i,t}\le\:{P}_{max}\:\:\forall\:t$$

where:

*P*_max_: The maximum allowed charging power across all ESVs at any time step.

Apart from these, the model also defines the limitations on the time period when the ESV can be charged and the charge strategy during the weekend based on the weekend working hours of individual ESVs.

**Fig. 2:**
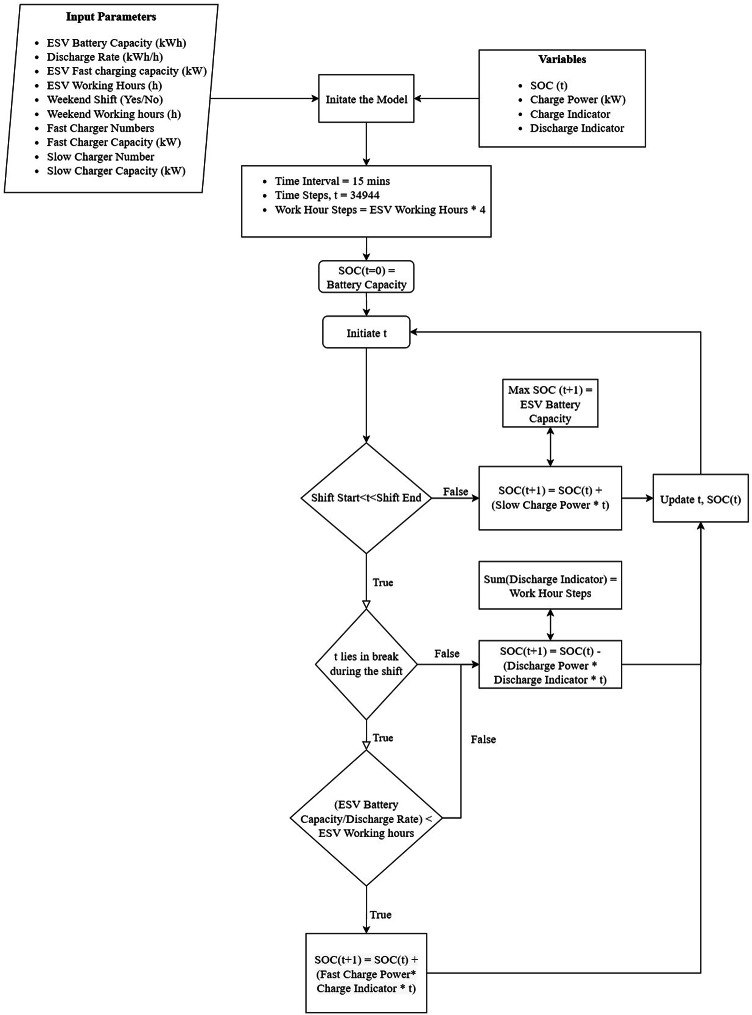
Flowchart for the optimization model.

A flowchart for the Optimization model is given below in Fig. 2:

#### Load management startegy with solar PV integration

Renewable energy sources like wind and solar can be used as an alternate source of energy in the grid. In this work, the potential of solar PV is explored. By integrating solar PV into the grid, the dependence on the external grid for energy import is reduced, thus reducing the overall strain on the grid. The hourly PV production profile can be created for a year based on the location and the PV system sizing. In the case of Port of Oskarshamn, the PV profile was shared and based on the solar yield factor, the PV system sizing was estimated.

##### Analysis

The results are analyzed in terms of transformer loading and line loading. The safe limit for both transformer and line loading is considered to be 80%^20^, reflecting a conservative operational threshold typically used in power system planning to account for thermal margins, load uncertainties, and equipment longevity. A similar margin of 80% is also considered for line loading^21^. The analysis focuses on the avoided or extended upgradation of network capacity through the implementation of mitigation measures.

## Case study

The case study is based on the Port of Oskarshamn, located on the southeastern coast of Sweden. The port is owned and operated by Smålandshamnar AB, while Oskarshamn Energi serves as the local grid operator. As a result, Oskarshamn Energi provided the necessary grid data and oversaw the implementation of the study.

### Grid design

**Fig. 3:**
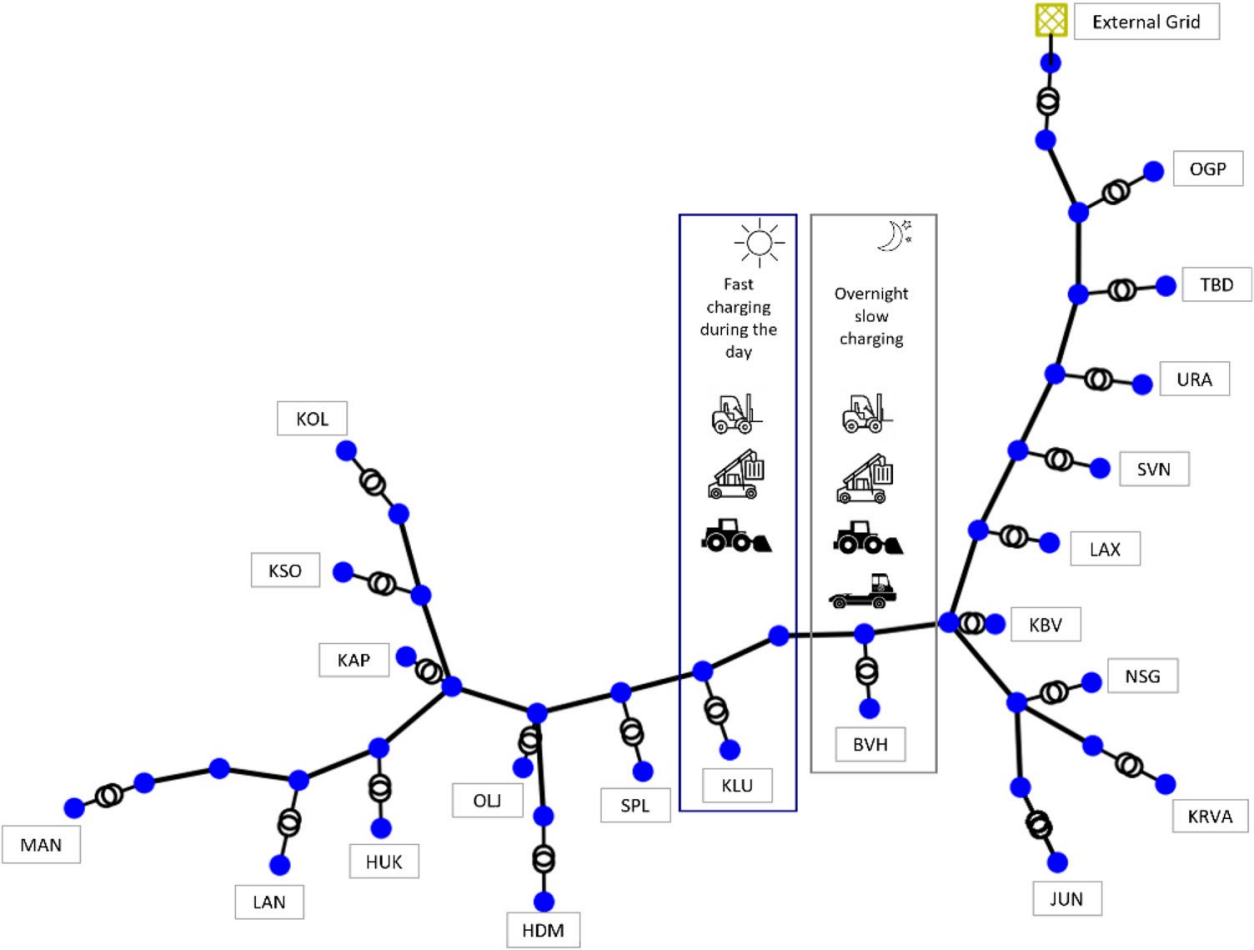
Grid network.

The grid network at the Port of Oskarshamn is represent as given in Fig. 3. All the components of the grid are designed as per the specifications. The data was shared by Oskarshamn Energi.

- **Buses**: The bus ‘EG’ at the substation is rated at 50 kV. All the buses at the primary end of the transformers in the network are rated at 10 kV, while the buses at the secondary are rated at 0.4 kV. The list of buses and corresponding rating is given in Table 1.
- **Lines**: An aluminium cable with the nomenclature ‘ACJJ_3 × 150 12 kV’ is used for the grid interconnection at the Port. The resistance (R), reactance (X), and capacitance (C) per km for cables are 0.21 Ω/km, 0.09 Ω/km, and 0.35 nF/km, respectively and the lengths are as given in Table 1.
- **Switches**: Some of the transformers are disconnected from the grid and the disconnection is represented by a switch. The switches between Bus_13 and Line_7, Bus_13 and Line_19, Bus_31 and Line_16, and Bus_35 and Line_18 are all open (state = FALSE) and have zero impedance (z_ohm = 0).
- **Transformers**: There is one transformer connected to the external grid and 30 transformers connected to the utilities in the grid. All the transformers connected to the utilities are rated at 10 kV at the primary and 0.4 kV at the secondary, while the transformer connected to the external grid is rated at 50 kV at the primary and 10 kV at the secondary. The apparent power rating of the transformers with other specifications are as per the Table 1. The short-circuit impedance (vkr), no-load loss (Pfe), and no-load current (i0) for transformers are 0.41%, 14 kW and 0.07% respectively.
- **Loads**: All the loads are connected at the secondary side of utility transformers. The real and apparent powers are considered in the model.

**Table 1:** Network specifications.

Component	Name	From	To	Length (km)	Max Current (kA)	Rated Power (MW)	High Voltage (kV)	Low Voltage (kV)	Short-Circuit Voltage (%)
Cable	Line_0	Bus_0	Bus_1	0.375	0.265	-	-	-	-
Cable	Line_1	Bus_1	Bus_3	0.081	0.265	-	-	-	-
Cable	Line_2	Bus_3	Bus_5	0.334	0.265	-	-	-	-
Cable	Line_3	Bus_5	Bus_7	0.66	0.265	-	-	-	-
Cable	Line_4	Bus_7	Bus_9	1.011	0.265	-	-	-	-
Cable	Line_5	Bus_9	Bus_11	0.393	0.265	-	-	-	-
Cable	Line_6	Bus_11	Bus_13	0.412	0.265	-	-	-	-
Cable	Line_7	Bus_13	Bus_15	0.143	0.265	-	-	-	-
Cable	Line_19	Bus_13	Bus_39	0.497	0.265	-	-	-	-
Cable	Line_8	Bus_11	Bus_17	0.297	0.265	-	-	-	-
Cable	Line_9	Bus_17	Bus_19	0.473	0.265	-	-	-	-
Cable	Line_10	Bus_19	Bus_21	0.558	0.265	-	-	-	-
Cable	Line_11	Bus_21	Bus_23	0.315	0.265	-	-	-	-
Cable	Line_12	Bus_23	Bus_25	0.441	0.265	-	-	-	-
Cable	Line_13	Bus_23	Bus_27	0.605	0.265	-	-	-	-
Cable	Line_14	Bus_27	Bus_29	0.729	0.265	-	-	-	-
Cable	Line_15	Bus_29	Bus_31	0.417	0.265	-	-	-	-
Cable	Line_16	Bus_31	Bus_33	0.646	0.265	-	-	-	-
Cable	Line_17	Bus_27	Bus_35	0.398	0.265	-	-	-	-
Cable	Line_18	Bus_35	Bus_37	0.366	0.265	-	-	-	-
Transformer	EXT	EG	Bus_0	-	-	25	50	12	4
Transformer	OGP	Bus_1	Bus_2	-	-	0.5	12	0.4	12
Transformer	TBD	Bus_3	Bus_4	-	-	0.5	12	0.4	12
Transformer	URA	Bus_5	Bus_6	-	-	0.5	12	0.4	12
Transformer	SVN	Bus_7	Bus_8	-	-	0.8	12	0.4	12
Transformer	LAX	Bus_9	Bus_10	-	-	1.6	12	0.4	12
Transformer	KBV	Bus_11	Bus_12	-	-	0.8	12	0.4	12
Transformer	NSG	Bus_13	Bus_14	-	-	0.8	12	0.4	12
Transformer	KRVA	Bus_15	Bus_16	-	-	0.8	12	0.4	12
Transformer	BVH	Bus_28	Bus_29			0.8	12	0.4	12
Transformer	KLU	Bus_33	Bus_34			0.8	12	0.4	12
Transformer	SPL	Bus_36	Bus_37			0.8	12	0.4	12
Transformer	OLJ	Bus_38	Bus_39			0.8	12	0.4	12
Transformer	HDM	Bus_40	Bus_41			0.8	12	0.4	12
Transformer	KAP	Bus_44	Bus_45			0.8	12	0.4	12
Transformer	HUK	Bus_46	Bus_47			0.8	12	0.4	12
Transformer	LAN	Bus_48	Bus_49			0.8	12	0.4	12
Transformer	MAN	Bus_51	Bus_52			0.5	12	0.4	12
Transformer	KSO	Bus_53	Bus_54			0.8	12	0.4	12
Transformer	KOL	Bus_55	Bus_56			0.8	12	0.4	12

### Baseline network state

The existing load in the network forms the baseline demand. For the model, the load is considered as a timeseries data at an interval of 15-minutes for 52 weeks, thus creating 34,944 timesteps. The year considered for the load data is 2022 based on the data availability with Oskarshamn Energi. After modelling all the componenents in the network using pandapower, the baseline demand was analysed to determine the status of the network.

### Scenario ideation

The scenario ideation is based on the Port of Oskarshamn’s plan to phase out conventional vehicles used in shore-side operations including forklifts, loaders, reach stackers, and terminal movers and replace them with electric counterparts. While the port’s decarbonization strategy remains unclear, the phase-out approach of conventional shore-side vehicles to replace it with electrified counterpart is aligned with the strategies adopted by the Port of Helsingborg^22^ and the Port of Södertälje^23^. As per the plan adopted by Oskarshamn Energi, the transition to ESVs follows a phased approach over two, five, and ten years. These timeframes form the basis for scenario ideation, referred to as the 2-year, 5-year, and 10-year scenarios. In the 2-year scenario, 22 conventional vehicles are replaced with ESVs. An additional five ESVs are introduced in the 5-year and 10-year scenarios, bringing the total number of ESVs to 27 and 32, respectively. The working hours of the newly deployed ESVs are modelled based on the operational patterns of their conventional counterparts, which range from 2 to 11 h per shift.

Charging strategies for the ESVs are designed to ensure that no additional vehicles are required to compensate for their operational hours. All ESVs undergo overnight slow charging, while fast charging requirements are determined based on the working hours in a shift.

### Load profile development

The development of the load profile is influenced by the daily operational hours and specifications of the Electric Shoreside Vehicles (ESVs). Typically, the port authority oversees and manages shoreside vehicle operations, and data related to these vehicles is usually sourced from them. However, in this case, data on the operating hours of existing conventional shoreside vehicles and the planned electrification was provided by the grid operator, Oskarshamn Energi.

Load profiles were created assuming that the ESVs follow similar working hours as the current conventional vehicle fleet. These profiles were developed for each scenario based on the cumulative ESV load. All ESVs are assumed to undergo overnight slow charging using individual 22 kW chargers, available from 19:30 to 07:00 the following day.

Fast charging is subject to certain constraints. Fast chargers are available only during scheduled breaks: 09:00–10:00 (morning), 11:45–13:00 (lunch), and 15:00–16:30 (evening). Each fast charger unit has three charging ports, allowing up to three ESVs to be charged simultaneously, with a total maximum capacity of 400 kW. The number of fast chargers varies by scenario: two in the 2-year scenario and three in the 5- and 10-year scenarios.

ESV specific data including battery capacity, charging and discharging rates, daily working hours, and weekly operational days contributed to developing the load profiles. A detailed mapping of which conventional shoreside vehicle is replaced by which ESV model was also shared. The specific combination of ESVs used in each scenario significantly influenced the resulting load profile. ESV specifications are summarized in the Table 2 below.

**Table 2:** ESV specification.

Model	Category	Battery Capacity (kWh)	Discharge Rate (kWh/h)	Fast Charge Capacity (kW)
Merlinum EA 6–600	Forklift	64	14	128
SMV 16 Ever	Forklift	110	20	147
Volvo L120H	Loader	237	47.5	158
SMV 46/32 E-ver	Reach Stacker	276	50	276
ERG450-65CS	Reach Stacker	587	115	587
Terberg RT 223 EL.	Terminal Mover	150	22.5	200

### Charging strategies

**Fig. 4:**
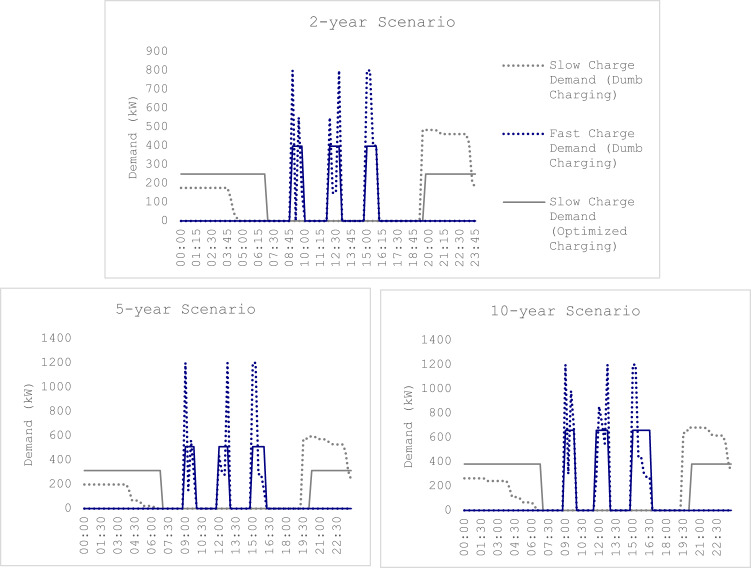
Scenario-wise comparison of fast charge and slow charge demands for dumb charging and load Management Strategy.

The daily load profile for load management strategy against dumb charging is as shown in the Fig. 4 below:

#### Dumb charging

The ESV load profile while adopting dumb charging does not consider the grid impact at the time of charging. Dumb charging strategy always utilizes the maximum charging capacity available at a timestep. With the help of the data for the ESV specification as in Table 2 and the working hours of the conventional shore-side vehicle, daily ESV demand profile is created for all the scenarios.

The fast charge and the slow charge demands are represented by blue and grey dotted lines in the graph respectively under Fig. 4. A 2-year scenario, with 22 ESVs and 10 of them requiring fast charging, utilizes the maximum charger capacity, both for fast charging and slow charging. The demand attained during each fast-charge session in the morning is 800 kW, which is the maximum installed charger capacity at 100% utilization and the slow-charge demand at 19:30 is 484 kW, implying all the 22 ESVs being charged at a maximum charge slow charge capacity of 22 kW. The peak demand however reduces at around 21:00, when some of the ESVs are fully charged and disconnected from the charger, reducing further for the subsequent time periods. A similar trend can be observed for the 5 and 10-year scenarios where the peak demand is 1200 kW since both scenarios have 3 fast chargers. The slow-charge demand, at 594 kW and 704 kW for 5 and 10-year scenarios, also utilized the maximum charge capacity.

#### Load management strategy

The load management strategy for the case of ESVs in the Port of Oskarshamn is driven by the algorithm as given in Sect. “Load management strategy”. The working hour and the ESV specific data are utilized to build the model for load management strategy. Through a better utilization of charger availability and regulation in charging demand, the objective of minimizing the maximum charging demand at a timestep is attained by the model.

As a result, the maximum demand attained by the model in 2-year scenario is 400 kW during fast charging and 249.5 kW during slow charging, translating to roughly a 50% reduction in demand as compared to dumb charging, as seen in Fig. 4 represented by solid lines. Similarly, at 510 kW and 313 kW for fast-charging and slow charging respectively, the charging demand in 5-year scenario also reduced significantly. Although the fast-charging and slow-charging demand further increases in a 10-year scenario load management strategy, at 660 kW and 382 kW, the demand is considerably lower as compared to dumb charging strategy.

#### With solar PV

With an aim to mitigate the impact of ESV on the electricity grid, solar PV was integrated into the model. The generation of the solar PV system, however, is limited by seasonal variation owing to the region’s high latitude. The energy generation during the months November to February is low due to limited hours of sunlight, while the summer months between May and August, the solar PV system can benefit from extended daylight hours. With the total solar energy production value and a yearly solar yield factor of 890 kWh/kWp as considered an average for Sweden^24^, the estimated solar PV system size is 2400 kW.

## Assumptions

- Reactive power was estimated using an assumed average power factor of 0.95 for all the transformers in the grid.
- For transformers with partially missing load profile data, yearly average values were used, while for those without any load profile data, demand was estimated based on transformers with similar kVA ratings.
- When converting hourly demand data to 15-minute intervals, it was assumed that demand remained constant across each time step within the hour.
- Charging and discharging efficiencies were considered 100%, meaning energy losses during these processes were not accounted for.
- The maximum state of charge (SOC) during charging was assumed to be equal to the battery capacity, while the minimum SOC during discharge was set at 5% of the battery capacity.
- The charging pattern was assumed to be linear across all SOC ranges, and no seasonal variations were considered.

## Results and discussion

This section discusses the impact of electrifying mobile utilities in the port for each charging strategies considering three scenarios; 2-, 5- and 10-year scenario, each representing a certain number of ESVs.

### Baseline network state

**Fig. 5:**
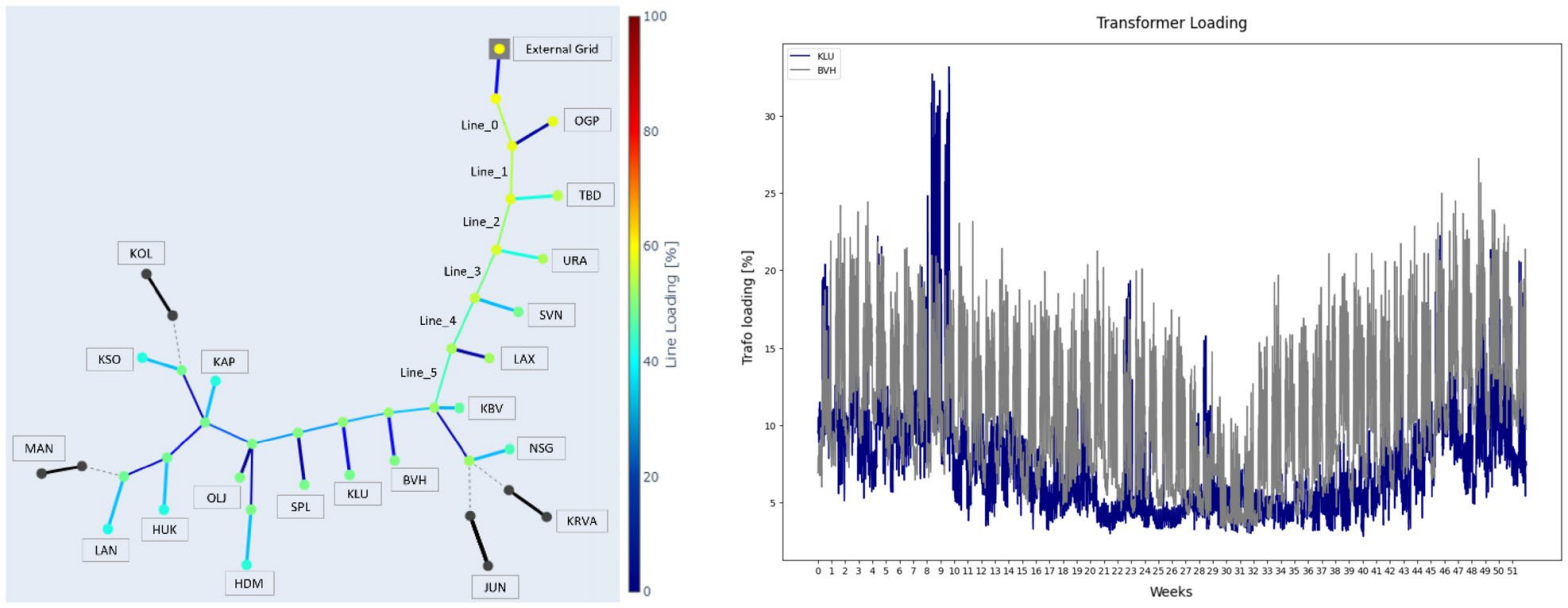
Line demand distribution in the network (left), annual demand of KLU and BVH in base scenario(right).

The baseline scenario consists of the existing grid without the addition of ESV charging demands. The grid was analysed for line and transformer loading to determine the initial loading percentages. All the transformers in the grid are loaded within their safe thresholds. Specifically, KLU and BVH, the transformers where fast and slow chargers will be connected are loaded at a maximum 33% and 27% respectively during weeks 8 and 9 as shown in Fig. 5. Similarly, line loading is analysed for different lines in the grid. Line_0, as seen in Fig. 5, experiences the highest stress in the grid since it connects the network to the external grid. The demand in the grid is solely satisfied by the external grid in this network and hence the current required to satisfy all the transformer demands passes through this line. The maximum line loading at Line_0 is 57% appearing during week 50, with decreasing loading percentage on the subsequent lines.

**Fig. 6:**
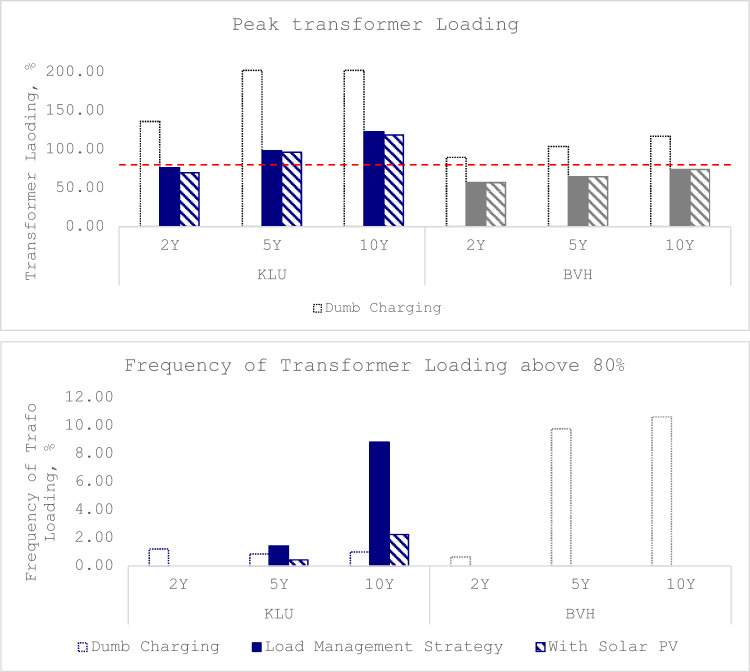
Peak transformer loading and transformer overloading graphs.

The impact of different charging strategies on the grid was evaluated across three scenarios: 2-year, 5-year, and 10-year, each representing an increasing number of Electrified Shore-side Vehicles (ESVs). The transformer parameters across all the different scenarios and charging strategies are as given in the Fig. 6. The dumb charging strategy led to significant overloading issues, particularly in the 5-year and 10-year scenarios, as represented by the dotted bar under the peak transformer loading graph. In the 2-year scenario, the KLU transformer reached a maximum loading of 135% during week 8, and the BVH transformer reached 89% during week 48, both exceeding the safe threshold of 80%. While the frequency of overloading was less than 5% of the time, Line_0 reached 76% loading, indicating increasing stress. In the 5-year scenario, the KLU transformer loading increased to 202%, and the BVH transformer to 104%, with lines 0, 1, and 2 exceeding safe thresholds, necessitating immediate network upgrades. In the 10-year scenario, the peak KLU transformer loading remains same at 202%, while the loading at KLU increases to 119%. The lines 0, 1 and 2, as in the previous scenario, exceeded the safe thresholds, confirming the need for grid upgrades.

In contrast, the load management strategy, as represented by the solid bar in Fig. 6, significantly reduced the loading and frequency of overloading. For the 2-year scenario, the KLU transformer loading decreased to 76%, and the BVH transformer to 56%, with no overloading and Line_0 loading at 65.5%. In the 5-year scenario, the KLU transformer loading increased to 97%, slightly exceeding the safe threshold, but the BVH transformer remained within limits. Line_0 loading was 69.7%, and no other lines exceeded safe thresholds. In the 10-year scenario, the KLU transformer loading increased to 123%, with a frequency of overloading of 8.8%, while the BVH transformer remained within limits. Despite the slight overloading of KLU, no immediate network upgrades were required for the 2-year and 5-year scenarios, but the 10-year scenario indicated a need for upgrades.

The integration of solar PV further enhanced the load management strategy, reducing the frequency of overloading and optimizing daytime demand as represented by the striped bar in the graphs under Fig. 6. In the 2-year scenario, the KLU transformer loading decreased to 69%, and the BVH transformer remained at 56%, with no overloading and Line_0 loading at 65.5%. For the 5-year scenario, the KLU transformer loading was 96%, with a frequency of overloading of 1.44%, and the BVH transformer remained within safe limits. Line_0 loading was 69.7%, and no other lines exceeded safe thresholds. In the 10-year scenario, the KLU transformer loading was 118%, with a reduced frequency of overloading of 2.3%, and the BVH transformer loading was 74%. Line_0 loading was 73.6%, and no other lines exceeded safe thresholds. The integration of solar PV and load management strategies significantly mitigated the impact of ESV loads, making it possible to accommodate more ESVs without immediate network upgrades in the 2-year and 5-year scenarios, although the 10-year scenario still indicated a need for upgrades. Table 3 provides a summary of network parameters across all scenarios and charging strategies.

**Table 3:** Summary of the network parameters across all the scenarios and charging strategies.

Scenario	Charging Strategy	KLU Loading (%)	BVH Loading (%)	Frequency of Overloading (%)	Line_0 Loading (%)	Other Lines Overloading	Need for Network Upgrade
2-Year	DC	135 (Wk 8)	89 (Wk 48)	KLU: <5%, BVH: <5%	76	No	Yes
	LM	76	56	No overloading	65.5	No	No
	PV	69	56	No overloading	65.5	No	No
5-Year	DC	202 (Wk 8)	104 (Wk 48)	KLU: 1%, BVH: 10%	87, 86.6, 82.8	Yes (Lines 0, 1, 2)	Yes
	LM	97 (Wk 8)	64	KLU: 1.5%	69.7	No	No
	PV	96	64	KLU: 1.44%	69.7	No	No
10-Year	DC	202 (Wk 8)	119 (Wk 48)	KLU: 1%, BVH: 11%	87, 86.6, 82.8	Yes (Lines 0, 1, 2)	Yes
	LM	123 (Wk 9)	74 (Wk 49)	KLU: 8.8%	73.6	No	Yes
	PV	118 (Wk 9)	74	KLU: 2.3%	73.6	No	Yes

### Discussion

The results of this study illuminate critical aspects of integrating Electric Shore-Side Vehicles (ESVs) into port operations and the effectiveness of proposed mitigation strategies.

Firstly, our detailed quantitative analysis of ESV load impacts on the local port grid demonstrates the substantial stress that unmanaged (‘dumb’) charging places on infrastructure. Key findings include the significant strain caused by these loads. Specifically, the analysis revealed that under ‘dumb’ charging strategies, transformer loading at critical points often exceeded the safe operational threshold. For instance, Transformer KLU (serving fast-charge loads) experienced peak loading that exceeded safe limits in all scenarios considered. Notably, in the 10-year projection scenario featuring the highest number of electrified vehicles, this method of charging posed challenges even with the advanced equipment, large-scale deployment indicating significant infrastructure upgrades might be required. The frequency of these dangerous loading peaks is also critical: in the 2-year scenario, transformer loading peaks occurred in less than 5% of the time annually. However, this frequency increases substantially with more vehicles connected, shooting up to 10% of the time in the annually projected 10-year scenario. Similarly, Transformer BVH (serving slow-charge loads), also experienced increased loading frequency, culminating in about 10% of the time crossing safe thresholds by the 10-year projection phase. Furthermore, the study records observed potential for line loading to exceed safe limits specifically in the 10-year projection year. This quantitative evidence underscores the necessity of implementing mitigation strategies to manage this load effectively.

Secondly, the evaluation of optimized charging strategies and solar PV integration demonstrates their practical efficacy. Our analysis illustrated that implementing optimized charging resulted in a significant reduction in overall grid stress compared to the unmanaged scenario. Notably, in the 2-year scenario, *all* grid parameters remained within safe thresholds when using optimized charging, thereby completely negating the immediate need (within two years) for substantial grid upgrades like the anticipated transformer replacement scheduled for Year 3. Regarding specific transformer impacts: Transformer KLU (fast charge loads) did show an increase in peak loading compared to the 2-year scenario when projecting up to the 5-year and 10-year mark. Since maximum charger capacity is not utilized after optimization, demand and thus transformer loading increase from the 5- to 10-year scenario. Critically, even as load demands increased with more ESVs (5-year and 10-year scenarios), the optimized strategy significantly mitigated the peak demand impacts compared to the unmanaged approach. While peak transformer loading at KLU breached the safe threshold by the 5-year scenario (where loading increased moderately but remained consistently high ~ 10% of the time), proposing upgrades there might be necessary, but if the primary concern transitions to frequency of overloading events, the analysis suggests focusing upgrades on Transformer BVH. This is because, although also breached with the dumb charging, the optimized charging scenario still resulted in a higher frequency of threshold crossings, i.e., 10% of the time, on Transformer BVH (containing slow charging) compared to KLU in the 10-year projection scenario under optimized charging. Therefore, decision-making prioritizing frequency mitigation points towards upgrading prospects for Transformer BVH rather than solely KLU, which possesses a substantial buffer capacity reducing threshold crossings but still contributes potential danger. The implementation of optimized charging was successful in maintaining Transformer BVH and associated line loadings below the safe threshold across all projected years, showcasing a robust capability of the strategy. Furthermore, the integration study revealed that adding a 2400 kW solar PV system, particularly beneficial for fast charge demands aligning with daytime peak production, helped manage infrastructure loads, reducing overloading frequency (a 35–40% frequency of transformer operating near zero demands indicating potential for energy storage and enhancing grid stability and energy independence. These quantitative results provide actionable insights for port operators and grid planners balancing electrification goals with grid reliability constraints.

## Conclusion

This study has successfully investigated the technical impacts of integrating Electric Shore-Side Vehicles (ESVs) into the Oskarshamn port grid infrastructure and assessed the effectiveness of combined mitigation strategies. Key results indicate that unmanaged charging significantly stresses the network, breaching transformer safety thresholds frequently (up to 10% of the time in projections) and necessitating near-term upgrades. In contrast, optimized charging strategies effectively reduced grid stress across projected growth phases (2-year, 5-year, 10-year), preventing transformer overload peaks in the shorter term and demonstrating a concrete ability to postpone substantial infrastructure upgrades, effectively deferring required investment from potentially year two to at least year five under the scenarios modelled. Furthermore, integrating solar photovoltaic generation demonstrated benefits in managing peak loads, reducing the frequency of overloading events, and enhancing grid stability and energy independence for the port.

However, this research has certain limitations. It primarily relied on model predictions based on representative operational data rather than continuous real-world monitoring, which inherently limits the perfect fidelity of correlation between simulated and real-world impacts, although the data points such as ‘2–11 hrs/day’ were based on real operational context analysis plan at the port. The model focused on specific network areas (substations KLU and BVH), and while providing significant insights, broader network impacts or effects stemming from incorporating more detailed real-world context factors were not extensively explored. Furthermore, the scope of this study intentionally centred on analysing technical performance and energy system impacts, thereby not delving deeply into associated cost implications or rigorous socio-behavioural analyses of charging decisions often required by analysts working on implementation strategies, which these authors recognise are crucial components for practical deployment. Addressing the identified limitations points towards valuable future research directions. A distinct future study is recommended to focus solely on an in-depth analysis of cost implications, including infrastructure, operational costs, and potential societal levies, alongside performance validation of cost rollout strategies using real-world data where available. Such focused research would complement the findings of this paper by providing a more holistic understanding of the economic and practical feasibility of decarbonising port utility operations.

## Data Availability

The datasets used and/or analysed during the current study are available from the corresponding author on reasonable request.
